# Rapid progression of marginal zone B-cell lymphoma after COVID-19 vaccination (BNT162b2): A case report

**DOI:** 10.3389/fmed.2022.963393

**Published:** 2022-08-01

**Authors:** Akinori Sekizawa, Kenichi Hashimoto, Shinichi Kobayashi, Sawako Kozono, Takahiro Kobayashi, Yusuke Kawamura, Motohiro Kimata, Naoya Fujita, Yosuke Ono, Yasuhiro Obuchi, Yuji Tanaka

**Affiliations:** ^1^Department of General Medicine, National Defense Medical College, Tokorozawa, Japan; ^2^Department of Hematology, National Defense Medical College, Tokorozawa, Japan; ^3^Department of Integrative Physiology and Bio-Nano Medicine, National Defense Medical College, Tokorozawa, Japan

**Keywords:** mRNA vaccine, B cell, lymphoma, COVID-19, lymphadenopathy, BNT162b2, marginal zone B-cell lymphoma

## Abstract

B-cell lymphomas are neoplastic diseases occasionally associated with chronic inflammation. mRNA vaccines for coronavirus disease 2019 (COVID-19) induce inflammatory responses, which often lead to fever and lymphadenopathies indistinguishable from lymphomas. Although both lymphadenopathies and lymphomas can be influential, the correlation between them is unclear. Herein, we present the first case of marginal zone B-cell lymphoma following mRNA COVID-19 vaccination. An 80-year-old Japanese woman presented with a right temporal mass that appeared the morning after she was administered her first mRNA COVID-19 vaccination (BNT162b2). The mass gradually decreased in size but persisted over 6 weeks after her first vaccination (3 weeks after her second vaccination). At her first visit to our hospital, ultrasound revealed the size of the mass to be 28.5 × 5.7 mm, and computed tomography revealed multiple lymphadenopathies in the right parotid, submandibular, jugular, and supraclavicular regions. Initially, we suspected head-and-neck benign lymphadenopathy as a side effect of vaccination. Nine weeks later, the number of swollen submandibular and parotid glands increased, and the lymph nodes further enlarged. Finally, the right temporal mass was diagnosed as marginal zone B-cell lymphoma based on immunohistochemical and flow cytometry findings of biopsy specimens. Our findings suggest that although 4–6 weeks of observation for lymph node inflammation after the second vaccination is recommended, malignancy should also be considered in the differential diagnosis of lymphadenopathy following vaccination.

## Introduction

During the coronavirus disease 2019 (COVID-19) pandemic, the mRNA COVID-19 vaccines, BNT162b2 and mRNA-1273, were administered worldwide. The clinical trials of the mRNA COVID-19 vaccines reported them as safe for administration, and their relationship with malignant diseases was rare (1, 2). Although a few cases of recurrence, progression, or regression of T-cell lymphoma after mRNA COVID-19 vaccinations were reported (3–5), an influence of these vaccines on B-cell lymphomas has not been reported to date.

In the abovementioned clinical trials, the incidence of lymphadenopathy after the administration of the BNT162b2 vaccine was 0.3% (1), while the incidence of lymphadenopathy after the administration of the mRNA-1273 vaccine was 6.1–11.6% (2). To avoid unnecessary invasive tests, such as biopsy of enlarged or hypermetabolic benign lymph nodes detected on ultrasound (US), computed tomography (CT), or ^18^F-fluorodeoxyglucose positron emission tomography-CT, close observation of vaccinated patients for inflammation of lymph nodes 4–6 weeks after the second dose (6) or at least 6 weeks after the booster dose (7–9) of COVID-19 vaccines is recommended. However, information about neoplastic lymphadenopathy derived from lymphoid hyperplasia following vaccinations remains unclear. Moreover, lengthy observations of clinical symptoms could lead to delayed diagnoses of malignant diseases, including fatal conditions.

Herein, we present a case of marginal zone B-cell lymphoma (MZL) in a patient after the administration of the BNT162b2 mRNA COVID-19 vaccine, which progressed during the 15-week follow-up from the first vaccination.

## Case description

**Figure 1:**
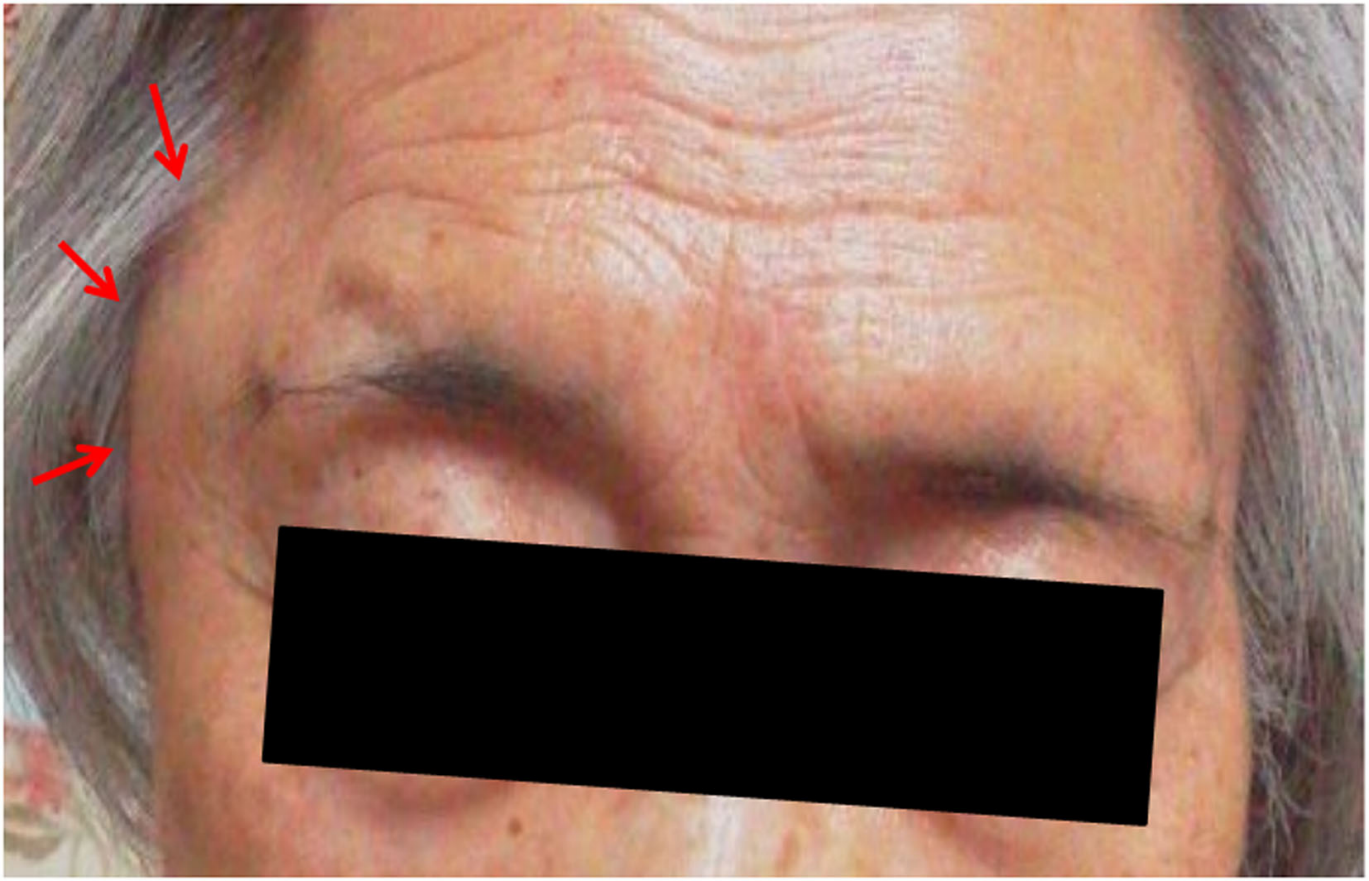
Photograph of the right temporal mass (red arrows) seen the morning after the patient's first coronavirus disease 2019 vaccination (BNT162b1) (photograph taken by the patient's family member).

An 80-year-old Japanese woman with a medical history of hypertension, angina pectoris, mitral valve regurgitation, and ovarian tumor resection was referred for further examination of a right temporal mass (Figure 1) that suddenly appeared the morning after she received her first COVID-19 vaccine (BNT162b2) in her left deltoid muscle. She had no fever, night sweats, weight loss, sticky sensation in her mouth, burning sensation in her eyes, or joint pain. Her elder sister had systemic lupus erythematosus, and her daughter had Sjögren's syndrome. She presented to her family doctor on the day that the mass first appeared and was prescribed acetaminophen and fexofenadine for this presumed side effect of the vaccination. The mass gradually decreased in size, but it did not disappear completely. She was asymptomatic except for a sore arm. Thereafter, she was administered the second vaccine. She was referred to our hospital 6 weeks after her first vaccination (3 weeks after her second vaccination) because the right temporal mass had not disappeared.

**Figure 2:**
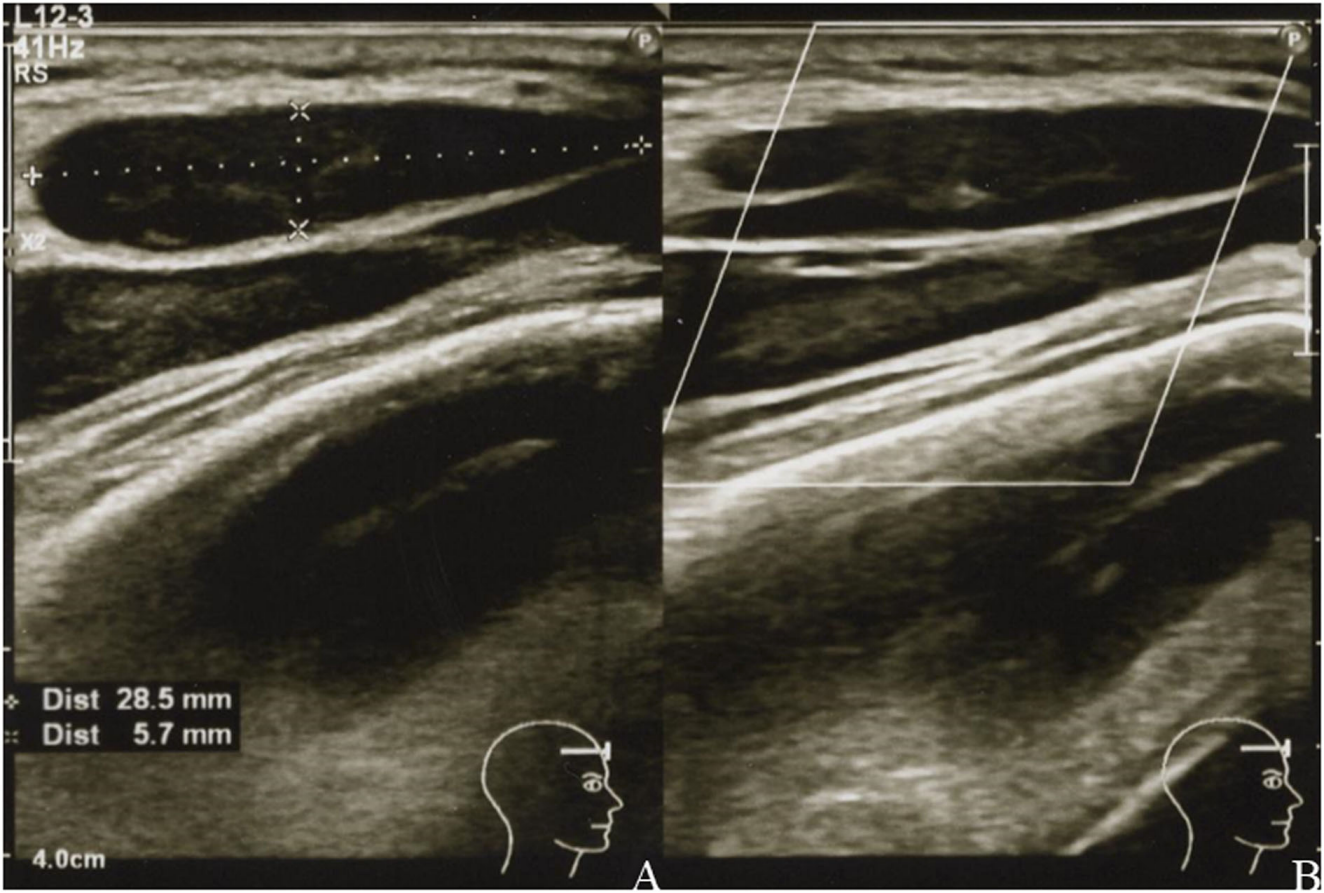
Grayscale **(A)** and color Doppler **(B)** images of the right temporal mass showing a uniformly hypoechoic teardrop-shaped mass with well-defined margins **(A)** and decreased vascularity **(B)**.

**Figure 3:**
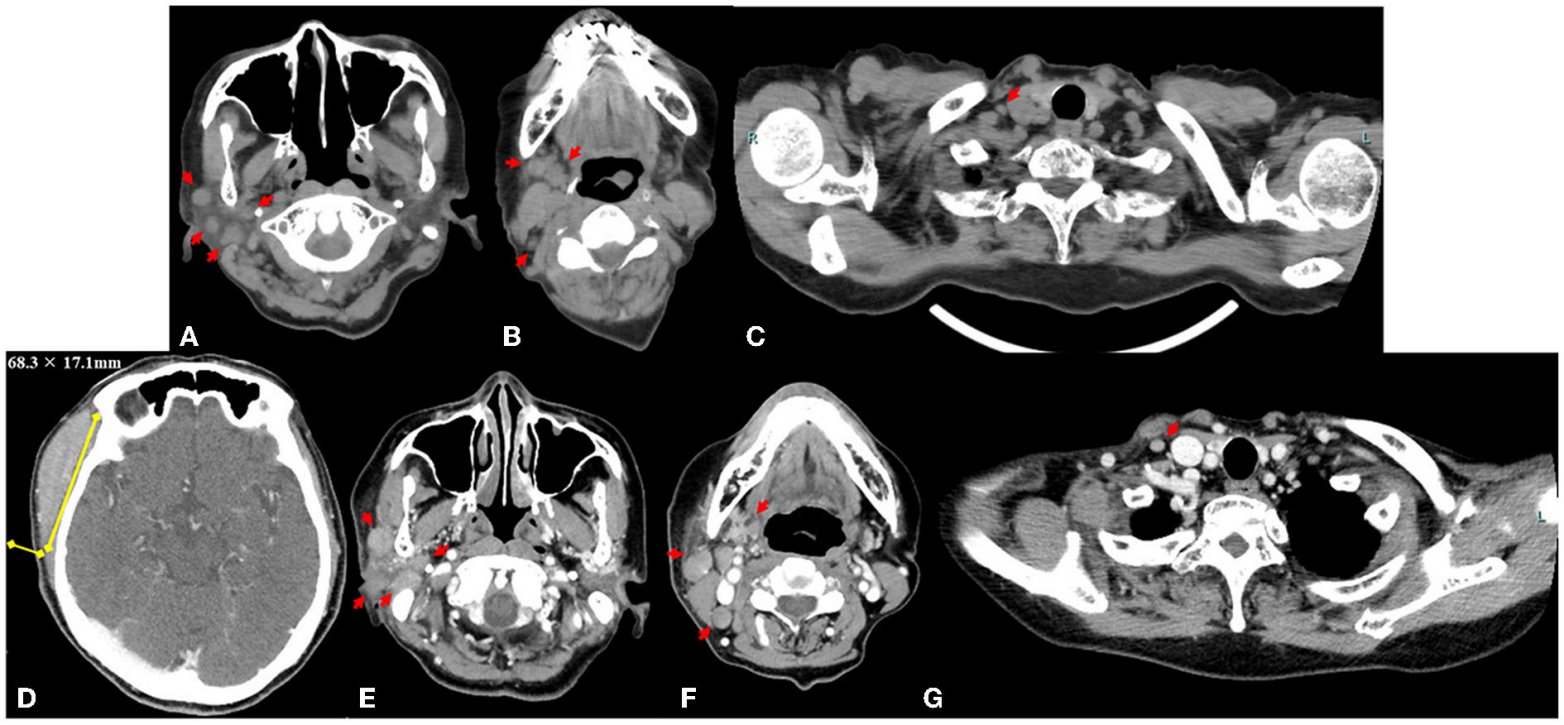
Axial computed tomography image obtained at the first visit shows 14 lymphadenopathies, with a maximum diameter ≤ 7.5 mm (red arrows) in the right parotid region **(A)**, submandibular and jugular regions **(B)**, and supraclavicular region **(C)**. Measurement of the right temporal mass (68.3 × 17.1 mm) on an axial contrast-enhanced computed tomography image 9 weeks after the first visit to our institution **(D)**. The lymphadenopathies (maximum size ≤ 13.3 mm, yellow arrows) in the right parotid region **(E)**, submandibular and jugular region **(F)**, and supraclavicular region **(G)**.

A physical examination identified a hard, immovable right temporal mass (~30.0 × 30.0 mm) and three palpable cervical lymph nodes. There was no tenderness or redness of the skin. US revealed the size of the mass to be 28.5 × 5.7 mm (Figures 2A,B), and the palpable lymph nodes were ≤ 10.2 mm in diameter. Laboratory test results of her blood and urine were unremarkable, including normal values of erythrocyte sedimentation rate and C-reactive protein, lactate dehydrogenase, and soluble interleukin-2 receptor levels. She tested negative for rheumatoid factor, SS-A and SS-B antibodies, and her antinuclear antibody titer was 1:40 (speckled pattern). CT of the neck, thorax, abdomen, and pelvis revealed 14 lymphadenopathies in the right parotid, submandibular, jugular, and supraclavicular regions, all of which were ≤ 7.5 mm (Figures 3A–C). At this time, the right temporal mass was suspected to be an asymptomatic benign lymphadenopathy after the administration of COVID-19 vaccination (BNT162b2). We decided on close monitoring without any treatment. We recommended that the patient return to our hospital if the mass increased in size or if she developed any other symptoms.

**Figure 4:**
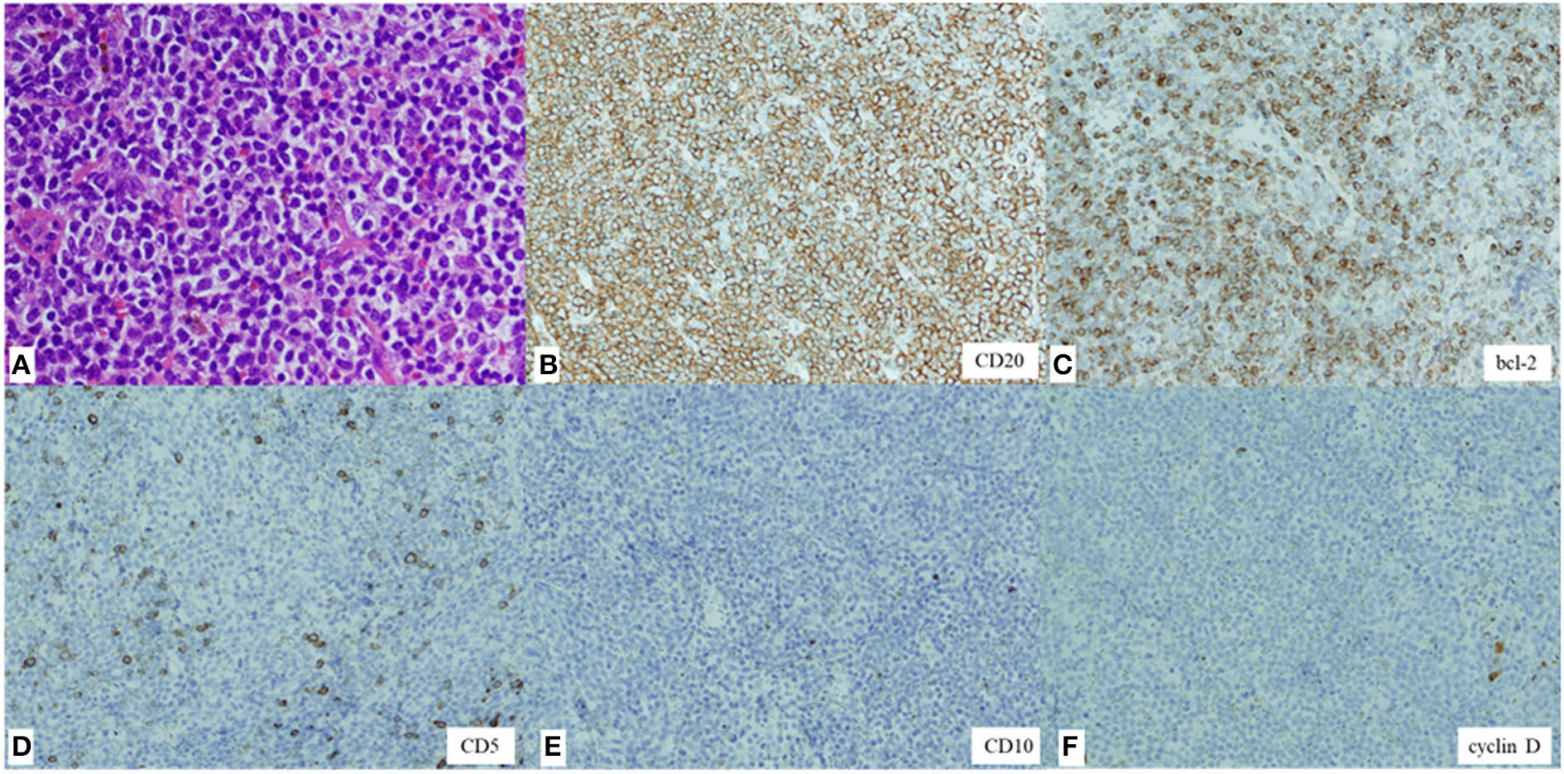
Hematoxylin and eosin staining of the biopsy specimen showing diffuse proliferation of small- to medium-sized lymphoid cells with slightly enlarged round nuclei (magnification, × 200) **(A)**. Immunohistochemical staining showing lymphoid cells positive for CD20 (× 100) **(B)** and bcl-2 (× 100) **(C)** and negative for CD5 (× 100) **(D)**, CD10 (× 100) **(E)**, and cyclin D (× 100) **(F)**.

Although the size of the mass did not change for 9 weeks after the first visit to our hospital (from weeks 6 to 15 after the first vaccination), she subsequently presented with a sudden enlargement of the mass over the course of a few days. Contrast-enhanced CT was performed again 15 weeks after the first vaccination. The size of the right temporal mass had increased from 28.5 × 5.7 mm to 68.3 × 17.1 mm in 10 weeks (from weeks 6 week 16 after the first vaccination) (Figure 3D). The number of swollen submandibular and parotid glands had also increased from 14 glands at the last visit to >22 glands, and the maximum size of the pre-existing lymphadenopathies had enlarged from 7.5 to 13.3 mm (Figures 3E–G). ^18^F-fluorodeoxyglucose (FDG) positron emission tomography-CT demonstrated abnormal FDG uptake in the mass, right parotid glands, and lymphadenopathies (maximum standardized uptake value, 6.92) (Supplementary Figure 1). A biopsy of the temporal mass was performed. Pathological examination showed diffuse proliferation of small- to medium-sized lymphoid cells with slightly enlarged round nuclei (Figure 4A). There were no indications of a lymphoepithelial lesion. In the immunohistochemical analysis, the lymphoid cells were positive for CD20 (Figure 4B), CD79a, and bcl-2 (Figure 4C) and negative for CD3, CD5 (Figure 4D), CD10 (Figure 4E), bcl-6, MUM1, cyclin D (Figure 4F), IgA, IgG, and IgM. In flow cytometry, the tumor cells were positive for CD19, CD20, CD22, and cyCD79a and negative for CD5, CD10, CD11c, CD23, CD25, and CD103. Immunoglobulin kappa light-chain restriction was detected (kappa: lambda ratio, 24:1). Therefore, the right temporal mass was diagnosed as a subtype of MZL (extranodal marginal zone lymphoma of the mucosa-associated lymphoid tissue or nodal marginal zone lymphoma) stage IIE, according to the Lugano classification (10). Careful observation or chemotherapy was suggested to the patient. The patient preferred careful monitoring over treatment for lymphoma and is now being followed up monthly as an outpatient. Because she had no symptoms, except difficulty in wearing glasses, and no functional abnormality in the liver, kidneys, or bone marrow, she was recommended watchful waiting for her lymphoma to avoid the risk of adverse effects of therapy. No significant changes in the size of the right temporal mass, which was 70.0 × 20.0 mm at the last follow-up, have been detected in the 10 months following the first vaccination.

## Discussion

To the best of our knowledge, this is the first report of B-cell lymphoma after mRNA COVID-19 vaccination, while three cases of T-cell lymphomas after mRNA COVID-19 vaccinations have been reported (3–5). Two of these three cases were of recurrence or progression of a CD30-positive T-cell lymphoma induced by mRNA COVID-19 vaccinations (3, 4), and one was of the spontaneous regression of a CD30-positive T-cell lymphoma (5). Although the precise mechanisms for T-cell lymphomas induced by the mRNA COVID-19 vaccines are still unknown, mRNA COVID-19 vaccines may have the capability to overstimulate the immune system as well as trigger autoimmune responses.

In our case, the same mechanism by which T-cell lymphomas are induced by the COVID-19 vaccine could be considered for the pathogenesis of MZL. mRNA COVID-19 vaccines are reported to induce T follicular helper cells with a Th1 functional profile, which is associated with selective generation of neutralizing antibodies, and stimulate germinal center B-cells, long-lived plasma cells, and memory B-cells. Therefore, these vaccines induce a stronger germinal center reaction than recombinant protein vaccines (11). However, the continuous stimulation of T- and B-cells by mRNA COVID-19 vaccines can trigger aberrant inflammatory responses, leading to lymphoma or accelerating its progression. MZLs are indolent lymphomas; their progression usually occurs over many years, and they are associated with chronic inflammation, including infections and several autoimmune diseases (12). A meta-analysis of five cohort studies reported that the standardized incidence rate of non-Hodgkin's lymphoma in patients with Sjögren's syndrome ranged from 8.7 to 44.4% (13). However, our patient showed no evidence of associated autoimmune diseases, including Sjögren's syndrome. A rare case has been reported in which the skin was induced by the antigenic stimulus of influenza vaccine components, causing MZL (14). A case of evolution of a cutaneous lymphoid hyperplasia, possibly due to a chronic inflammatory response to the antigenic stimulus of tattoo dye to a B-cell lymphoma, has also been reported (15). The pathogenesis of MZL in the present case could be similar to that in the abovementioned cases.

Imaging and clinical features characteristic of malignancy that are useful in differential diagnosis include >11 enlarged lymph nodes, lymph nodes with short-axis diameters >15 mm, lymphadenopathy on the contralateral side of the vaccination site, and/or lymphadenopathy in a region other than the axial and supraclavicular regions. Cervical lymphadenopathy after administration of mRNA COVID-19 vaccines is not rare, with a reported prevalence of 9% on CT (16) and 5 and 14.8% on US (17, 18). The number of cervical lymphadenopathies ranged from 1 to 10 on CT (16) and 1–5 on US (19). The median short-axis diameter of the largest node in the cervical lymphadenopathy was 7 mm (range, 5–14 mm) on CT (16) and 5.2 mm (range, 3.4–8.2 mm) on US (18). mRNA COVID-19 vaccine-related lymphadenopathy was mostly identified on the injected side (1, 2) and in the axial and/or supraclavicular regions (20, 21). Cocco et al. also reported that lymph node swelling after COVID-19 vaccination most commonly appeared in the axillary and/or supraclavicular regions; however, 5/64 (7.8%) patients in their study had swollen lymph nodes in an atypical location [6/170 (3.5%) lymph nodes] (22). In our case, most of the lymph nodes [13/14 (93%) at the first visit; 20/22 (91%) at the second visit] were in atypical locations, such as in the parotid, submandibular, and jugular regions. Therefore, cervical lymphadenopathy with >11 enlarged lymph nodes, lymph nodes with short-axis diameters >15 mm, and/or lymph nodes on the contralateral side of the injection site or in a region other than the axial and/or supraclavicular regions is considered atypical, and these clinical findings could be suggestive of a malignancy.

In our case, the patient's CT at the first visit showed 14 lymphadenopathies in the right parotid, submandibular, jugular, and supraclavicular regions on the contralateral side of the COVID-19 vaccination site. The mass was 28.5 × 5.7 mm in size, and the diameter of the largest lymph node was 7.5 mm on CT and 10.2 mm on US. Therefore, the number and location of the lymphadenopathies and the size of the mass were atypical for a vaccine-related cervical lymphadenopathy, which contributed to suspicion of malignant disease and further examinations.

The shape of the lymphadenopathy on US, findings of eccentric cortical thickness, irregular margins, loss of an echogenic hilum, and increasing peripheral vascularity on a color Doppler scan are generally suggestive of malignancy (23, 24). Of these findings, the mass in our case showed eccentric cortical thickness. However, many mRNA COVID-19 vaccine-related lymphadenopathies show asymmetric eccentric cortical thickening, loss of an echogenic hilum, and increased vascularity on US (18, 19). Therefore, other than the irregular margin, mRNA COVID-19 vaccine-related lymphadenopathies can be indistinguishable from neoplastic lymphadenopathies.

In a previous study, no significant differences in US findings were detected among patients with COVID-19 vaccine-related lymphadenopathies who received the Pfizer/BioNTech BNT162b2 mRNA, AstraZeneca ChAdOx1, and Moderna mRNA-1273 vaccines (25). In contrast, the US features of lymph nodes post-vaccination, such as round morphology, absence of hilum, and hard pattern, have been found to mimic those of pathological lymph nodes (25). However, in the patients in that study, features of COVID-19 vaccine-related lymphadenopathy were found to be superimposed on those of malignant pathologies, representing a potential pitfall for patients belonging to high-risk categories. Therefore, patients with COVID-19 vaccine-related lymphadenopathy should receive comprehensive care and follow-up.

Recommendations by the Canadian Society of Breast Imaging, Society of Breast Imaging, and European Society of Breast Imaging for the treatment of lymphadenopathy after the administration of mRNA COVID-19 vaccines advise waiting and monitoring for over 4–6 weeks (6, 8, 9). However, opinions differ on whether a long-term observation is acceptable for distinguishing benign from neoplastic lymphadenopathy because lymph node swelling after the administration of mRNA COVID-19 vaccines has been reported to persist for over 4 weeks in 50% (18) and 20% (26) of patients on US. In particular, lymphadenopathy in an atypical region (such as the upper cervical region), involvement of multiple lymph nodes, and extraordinary enlargement of lymph nodes may need to be observed for a shorter duration of ~4 weeks before treatment, as recommended by the three societies (6, 8, 9). On the contrary, lymphomas that are relatively benign and have a long progression (as seen in this case) pose a risk of misdiagnosis or a missed diagnosis if the lymphoma develops after vaccination. Therefore, we recommend careful observation of post-COVID-19 vaccination lymph node enlargements, even if they occur 4–6 weeks after the second vaccination.

In conclusion, we reported a case of MZL following mRNA COVID-19 vaccination. This is the first report of a malignant lymphoma of B-cell lineage that developed after COVID-19 vaccination. Lymphadenopathy induced by mRNA COVID-19 vaccination is not rare; therefore, clinicians should be aware of the atypical features of lymphadenopathy to prevent delayed diagnosis during monitoring of the signs and symptoms listed above. We suggest that malignant lymphoma may be differentiated from benign lymphadenopathy if there are >11 enlarged lymph nodes, lymph nodes with short-axis diameters >15 mm, and lymphadenopathy contralateral to the vaccination site and/or in a region other than the axial and supraclavicular regions. Attention should be paid to the development of lymphoma within 4–6 weeks after COVID-19 vaccination. Moreover, care should be taken to avoid overlooking relatively benign, slowly progressing lymphomas, such as MZLs, after 4–6 weeks of follow-up.

## Patient perspective

This patient wanted to notify the possibility of neoplastic lymphadenopathy mimicking lymphadenopathy following the mRNA COVID-19 vaccinations as a caution. Moreover, she wanted to utilize our therapeutic experience for other patients with COVID-19 vaccine-related lymphadenopathies to prevent negligence or delayed diagnoses.

## Data availability statement

The data that support the findings of this article/Supplementary material are available from the corresponding author upon reasonable request.

## Ethics statement

This study was reviewed and approved by the Ethics Committee at National Defense Medical College in Japan. The patient provided written informed consent to participate in this study. Written informed consent was obtained from the patient for the publication of any potentially identifiable images or data included in this article.

## Author contributions

AS and KH wrote the first draft of the manuscript and sections of the revised manuscript. SKob was consulted for the diagnosis and hematological problems. KH contributed to funding acquisition. AS, SKob, KH, SKoz, TK, YK, MK, NF, YOn, YOb, and YT were responsible for the clinical care of the patient and supervised the writing of the manuscript. All authors contributed to manuscript revision and read and approved the submitted version.

## Funding

This work was supported by the Fukuda Foundation 2019 for medical technology.

## Conflict of interest

The authors declare that the research was conducted in the absence of any commercial or financial relationships that could be construed as a potential conflict of interest.

## Publisher's note

All claims expressed in this article are solely those of the authors and do not necessarily represent those of their affiliated organizations, or those of the publisher, the editors and the reviewers. Any product that may be evaluated in this article, or claim that may be made by its manufacturer, is not guaranteed or endorsed by the publisher.
