# Supplementary material for: Rapid progression of marginal zone B-cell lymphoma after COVID-19 vaccination (BNT162b2): A case report
Source: Front Med (Lausanne). 2022 Aug 1;9:963393. doi: 10.3389/fmed.2022.963393 (PMC9377515; doi:10.3389/fmed.2022.963393)
Supplement: Supplementary file 1 [file Data_Sheet_1.docx]

Supplementary Material

# Supplementary Figures


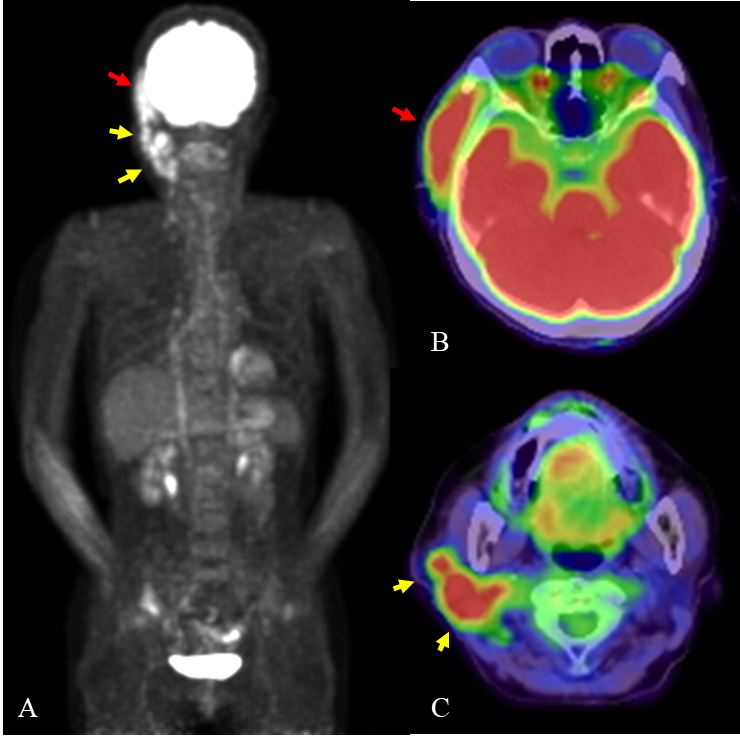


**Supplementary Figure 1.** Coronal maximum intensity projection image (A) and fused axial images (B, C) of 18F-fluorodeoxyglucose (FDG) positron emission tomography-computed tomography showing abnormal FDG uptake of the mass (red arrow), right parotid glands, and lymphadenopathies (yellow arrows) (maximum standardized uptake value, 6.92).
